# Ultrastructural Analysis of the Saphenous Vein Endothelium in a Patient With Proinflammatory Comorbidities Undergoing Coronary Artery Bypass Grafting

**DOI:** 10.1155/carm/9914323

**Published:** 2026-01-28

**Authors:** Matheus Duarte Pimentel, Heraldo Guedis Lobo Filho, José Glauco Lobo Filho

**Affiliations:** ^1^ Department of Surgery, Federal University of Ceará, Fortaleza, Brazil, ufc.br

**Keywords:** coronary artery bypass grafting, saphenous vein, scanning electron microscopy, vascular endothelium

## Abstract

The saphenous vein (SV) remains one of the most widely used grafts in coronary artery bypass grafting (CABG), and the integrity of the endothelium—a complex structure susceptible to deleterious effects from proinflammatory comorbidities—is a critical factor for graft patency. A 75‐year‐old patient with hypertension, dyslipidemia, insulin‐dependent Type 2 diabetes mellitus, gout, peripheral arterial occlusive disease, and active smoking presented with unstable angina and cardiogenic shock, exhibiting severe coronary artery disease refractory to medical treatment. Emergency CABG was indicated due to the patient’s clinical deterioration. During the procedure, the SV was harvested using skin‐bridged incisions in an atraumatic manner with minimal handling. Immediately after excision, the SV was maintained at room temperature (∼20°C), and a venous cannula was attached to the distal portion of a 3‐cm segment, which was perfused with a fixation solution containing 2.5% glutaraldehyde, 4% paraformaldehyde, and 0.1 M sodium cacodylate buffer (pH 7.4). It was then stored in an isothermal container and transported for scanning electron microscopy (SEM) analysis. SEM revealed significant endothelial damage, with extensive areas of endothelial cell detachment and loss, exposure of the basement membrane and collagen fibers, and—particularly in areas of endothelial denudation—the presence of fibrin aggregates and microthrombi. This case suggests that, in critically ill patients with multiple comorbidities, the SV may exhibit substantial endothelial damage, including microthrombi formation, immediately after surgical excision. These findings are associative and speculative; further studies are needed to explore whether such changes contribute to an increased risk of early venous graft failure. Upon identifying patients at high risk, intensive therapeutic measures should be promptly implemented to address comorbidities and minimize their deleterious effects on the endothelium.

## 1. Introduction

The saphenous vein (SV) remains one of the most widely used grafts in coronary artery bypass grafting (CABG), despite increasing recommendations for the use of arterial grafts beyond the left internal thoracic artery (LITA) [1, 2].

The integrity of the endothelium is a critical factor for the patency of vascular grafts, and in the SV, this structure is particularly susceptible to injury during surgical harvesting and preparation, mainly due to direct trauma, high distension pressures, and the use of nonbiocompatible preservation solutions—factors of significant pathophysiological relevance but relatively underappreciated during excision [3, 4].

The presence of comorbidities such as hypertension, diabetes mellitus, hypercholesterolemia, active smoking, and others that increase inflammatory activity has a direct impact on endothelial function and structure. Increased thrombogenicity, leukocyte adhesion, and abnormal vasoreactivity are some of the deleterious effects of these factors, which are not limited to the arterial endothelium [5–7].

The objective of this report is to describe the ultrastructural morphological changes observed in the SV endothelium in a critically ill patient with multiple comorbidities undergoing CABG.

## 2. Case Report

A 75‐year‐old patient with hypertension, dyslipidemia, insulin‐dependent Type 2 diabetes mellitus, gout, peripheral arterial occlusive disease, and active smoking presented with unstable angina and cardiogenic shock, exhibiting severe coronary artery disease (CAD) refractory to medical treatment. Emergency CABG was indicated due to the patient’s clinical deterioration.

During the procedure, the SV was harvested using skin‐bridged incisions in an atraumatic manner with minimal handling. Immediately after excision, the SV was maintained at room temperature (∼20°C), and a venous cannula was attached to the distal portion of a 3‐cm segment of the graft, which was perfused with a fixation solution containing 2.5% glutaraldehyde, 4% paraformaldehyde, and 0.1 M sodium cacodylate buffer (pH 7.4). The time interval between the venous segment excision and initiation of fixation was less than 5 minutes. The SV segment was then stored in an isothermal container and transported for analysis by scanning electron microscopy (SEM). The venous sample stored in the fixation solution was removed, washed three times in 0.1 M sodium cacodylate buffer, and dehydrated through a graded ethanol series (50%, 70%, 90%, and 100%). After dehydration, samples underwent critical point drying, were mounted on SEM stubs with carbon adhesive tape, and sputter‐coated with 20‐nm gold.

Imaging was performed using a Quanta 450 FEG scanning electron microscope (Thermo Scientific) at 10.0 kV. For the SV segment, the entire endothelial area was thoroughly examined at increasing magnifications (50×, 500×, 1000×, and 5000×) by two blinded observers.

SEM analysis revealed significant endothelial damage, with extensive areas of endothelial cell detachment and loss, exposure of the basement membrane and collagen fibers, and—particularly in areas of endothelial denudation—the presence of fibrin aggregates and microthrombi (Figure 1).

**Figure FIGURE 1:**
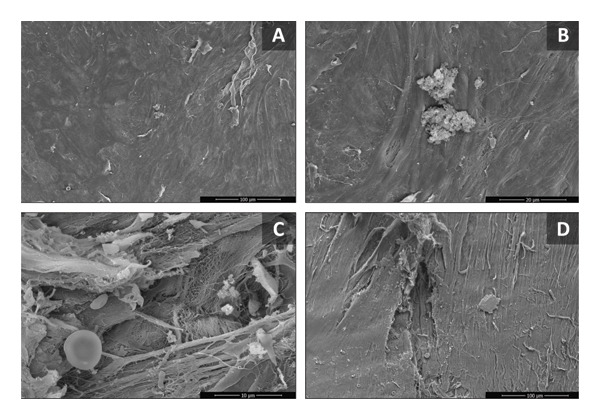
Scanning electron microscopy images of the luminal surface of saphenous vein segments from the patient. (A) Luminal surface with areas of detachment of the endothelial surface and loss of the integrity of the intimal layer. (B) Higher magnification of the endothelial surface illustrating a cluster of fibrin and platelets. (C) Detailing of an area of endothelial loss, with exposure of collagen fibers and deposition of platelets, red blood cells, and fibrin. (D) Extensive area of endothelial cell separation with exposure of the subendothelial layer.

These findings were not observed in SV segments from a control group of patients without comorbidities analyzed in a previous study by our group [8].

## 3. Discussion

The limited long‐term patency of the SV, with associated unfavorable clinical outcomes, remains one of the main limitations of its use in CABG. However, due to the ease of dissection and low morbidity associated with SV harvesting, this graft continues to be relevant, particularly in urgent situations where rapid graft acquisition is critical [1, 9].

Deleterious changes in the endothelium contribute to increased thrombogenicity, which may lead to early venous graft failure and, in the longer term, correlate with increased oxidative stress and inflammatory responses, accelerating the development of intimal hyperplasia and atherosclerosis [1, 7, 10].

Furthermore, the influence of proinflammatory factors on the venous endothelium differs from that observed in the arterial endothelium. Venous endothelial cells tend to respond to insults—whether acute or chronic—with significant leukocyte recruitment and expression of adhesion molecules such as P‐selectin, intercellular adhesion molecule (ICAM‐1), and vascular cell adhesion molecule (VCAM‐1) [7, 11]. These leukocytes directly damage the endothelium by expressing proinflammatory cytokines, also leading to increased platelet adhesiveness and reduced bioavailability of prostaglandins, particularly nitric oxide (NO) and hydrogen sulfide (H_2_S)—a gasotransmitter associated with antiatherogenic, antithrombotic, and vasodilatory properties [10–13].

Certain pathologies compromise endothelial structure and/or function, with diabetes mellitus, heart failure, and renal insufficiency being notable, as these are associated with early venous graft failure, in addition to dyslipidemia—a critical factor in the development of ischemic heart disease. Diabetic patients exhibit reduced cellular capacity to mitigate reactive oxygen species (ROS), as well as elevated serum levels of selectin, tissue plasminogen activator (t‐PA), von Willebrand factor (vWF), and C‐reactive protein (CRP) [5, 6]. This results in persistent proinflammatory stimulation, creating a proatherogenic environment in the arterial endothelium and a prothrombotic environment in the venous endothelium, reducing NO production and its ability to counteract the deleterious effects of superoxides (O_2_^-^) [6, 7, 11].

Heart failure, with increased venous congestion (also seen in patients with renal insufficiency), causes biomechanical stress on the endothelium [14]. Additionally, the neurohormonal activation typically involved in the pathophysiological spectrum of this disease results in the endothelium not only being affected by elevated serum cytokine levels but also serving as a primary site of their production [1, 9, 14, 15]. This was evidenced by Columbus et al., who demonstrated that acute venous tissue distension led to increased levels of interleukin‐6 (IL‐6), VCAM‐1, ICAM‐1, vWF, and endothelin‐1 (ET‐1) [15]. Hypercholesterolemia, with elevated serum low‐density lipoprotein (LDL) cholesterol levels, is also associated with increased O_2_^-^ formation, primarily through stimulation of enzymes such as NADPH oxidase and reduced activity of endothelial nitric oxide synthase (eNOS) [11, 15, 16].

It should be noted that surgical harvesting and the use of inadequate handling and preservation techniques for the SV, such as infusion of saline solution and high‐pressure intraluminal distension, lead to direct endothelial loss and activation of endothelial cells, with increased expression of the proinflammatory and prothrombotic molecules described previously [1, 3, 4].

The combination of an environment composed of vulnerable endothelial cells exposed to a chronically prothrombotic state may enable the insult of surgical harvesting to exacerbate endothelial damage, resulting in extensive areas of endothelial cell denudation—a factor directly related to early SV graft thrombosis [4, 9, 11].

Although SEM studies of SV endothelial injury are not new and have previously documented damage associated with graft harvesting, particularly in comparisons of conventional versus no‐touch or endoscopic techniques, most prior reports focus on procedural trauma in elective, lower‐risk patients. The present case adds incremental value by documenting extensive endothelial denudation with prominent microthrombi immediately after excision, i.e., before any implantation or exposure to arterial hemodynamics, in a critically ill patient with multiple proinflammatory comorbidities. This observation suggests that preexisting endothelial vulnerability, possibly driven by the patient’s comorbidities, may predispose to rapid, severe ultrastructural changes even with an atraumatic harvesting technique, potentially contributing to early graft failure beyond what is typically attributed to surgical manipulation alone.

Although our study has the inherent limitations of a case report and the difficulty in precisely characterizing the extent of SV endothelial damage, it is worth noting that ultrastructural descriptions of immediate postexcision changes in such high‐risk patients are scarce, making the findings described here relevant for better characterizing this pattern of cellular injury.

In brief, our observations suggest that, in critically ill patients, the SV may exhibit significant endothelial damage, including microthrombi formation, immediately following surgical excision. These findings are associative and based on data from a single case; therefore, they remain speculative and do not establish causation. Further studies are needed to determine whether such changes may contribute to an increased risk of early venous graft failure and to explore potential underlying mechanisms.

## Funding

No funding was received for this manuscript.

## Ethics Statement

We declare that the patient approved the study by signing an informed consent form, and the study followed the established ethical guidelines.

## Conflicts of Interest

The authors declare no conflicts of interest.

## Data Availability

The data that support the findings of this study are available from the corresponding author upon reasonable request.
